# A Novel Approach to Detect Drones Using Deep Convolutional Neural Network Architecture

**DOI:** 10.3390/s24144550

**Published:** 2024-07-13

**Authors:** Hrishi Rakshit, Pooneh Bagheri Zadeh

**Affiliations:** School of Built Environment, Engineering and Computing, Leeds Beckett University, Headingley Campus, Leeds LS6 3QS, UK; h.rakshit2399@student.leedsbeckett.ac.uk

**Keywords:** drone classifications, deep convolutional neural network, hyperparameters, drone audio signal, drone datasets

## Abstract

Over the past decades, drones have become more attainable by the public due to their widespread availability at affordable prices. Nevertheless, this situation sparks serious concerns in both the cyber and physical security domains, as drones can be employed for malicious activities with public safety threats. However, detecting drones instantly and efficiently is a very difficult task due to their tiny size and swift flights. This paper presents a novel drone detection method using deep convolutional learning and deep transfer learning. The proposed algorithm employs a new feature extraction network, which is added to the modified YOU ONLY LOOK ONCE version2 (YOLOv2) network. The feature extraction model uses bypass connections to learn features from the training sets and solves the “vanishing gradient” problem caused by the increasing depth of the network. The structure of YOLOv2 is modified by replacing the rectified linear unit (relu) with a leaky-relu activation function and adding an extra convolutional layer with a stride of 2 to improve the small object detection accuracy. Using leaky-relu solves the “dying relu” problem. The additional convolution layer with a stride of 2 reduces the spatial dimensions of the feature maps and helps the network to focus on larger contextual information while still preserving the ability to detect small objects. The model is trained with a custom dataset that contains various types of drones, airplanes, birds, and helicopters under various weather conditions. The proposed model demonstrates a notable performance, achieving an accuracy of 77% on the test images with only 5 million learnable parameters in contrast to the Darknet53 + YOLOv3 model, which exhibits a 54% accuracy on the same test set despite employing 62 million learnable parameters.

## 1. Introduction

The utilization of unmanned arial vehicles (UAVs), or drones, has grown significantly due to the escalating adoption of drones for both commercial and recreational purposes. As a result, the imperative of effective drone detection has become increasingly paramount. Drones can be used for a variety of purposes, including delivery of goods [1], photography [2], and even surveillance [3]. Drones play a multifaceted role in modern agriculture [4], industrial applications [5], building smart cities [6], monitoring weather conditions for safe driving [7], and many more applications.

Although drones are used for a multitude of applications, they can pose significant threats to public safety and security when they are used inappropriately or with malicious intent. A crucial apprehension related to drones is their potential use in terrorist attacks or other forms of violent activities. Drones can be equipped with a variety of payloads [8], including explosives or chemical weapons, and can be flown into sensitive areas or crowded locations to cause harm [9]. In addition to security concerns, if a drone were to collide with an aircraft during flight, the results could be catastrophic. Drones are used to send illegal drugs, communication devices, such as mobile handsets, and other illegal goods into prisons [10]. Spy drones are used to collect sensitive information [9]. It is necessary to detect drones in restricted areas as early as possible to prevent any fatal or devastating outcomes.

To ensure the preservation of public safety and privacy, as well as the prevention of potential terrorist activities, the timely detection of drones within restricted regions is vital. There are some systems that are used to detect drones in restricted areas, such as RADAR-based systems [11], radio-frequency analyzers [12], acoustic characteristic tracers [13], and visualization-based detection [14]. This paper discusses the merits and demerits of these existing systems and proposes a new algorithm for drone detection. The rest of the paper is organized as follows: in Section 2, existing state-of-the-art techniques for drone detection are presented; the datasets used for the training and testing are discussed in Section 3; Section 4 presents the proposed algorithm for drone detection; comparative simulation results are shown in Section 5; Section 6 evaluates the overall performance of the proposed algorithm, a general discussion of the novelties of the research is presented in Section 7; and, finally, Section 8 concludes the paper.

## 2. Literature Review

For decades, RADAR technology has served as a primary means of aerial vehicle detection. Nevertheless, conventional RADAR technology suffers from several limitations in detecting small UAVs or drones. Firstly, the flight speeds of drones are too fast to compare with the RADAR velocity and the size of the drones are too tiny to reflect any meaningful electromagnetic waves [11,12]. Another limitation is that RADAR cannot distinguish among other small flying objects, such as birds and drones [12].

Another popular technique is radio-frequency (RF)-based drone detection. This approach captures communications between drones and ground-controlled devices. However, in many cases, drones are automatically driven using on-board software rather than ground-controlled devices [11].

Using the acoustic characteristics of drones for their detection is a promising approach compared to other state-of-the-art methods, as drones have very unique acoustic characteristics than other small flying objects [13]. However, this method is not feasible in very noisy environments such as airports.

Recently, vision-based object detection methods have received significant attention by researchers due to advances in smart vision technology. There are two types of visualization-based object detection systems, as follows: one is traditional computer vision-based detection systems, and the other is deep learning-based detection systems.

Traditional computer-vision-based detection methods, such as histogram of oriented gradients (HOG) [13] and scale-invariant feature transform (SIFT) [14], depend on handcrafted features, which may not be robust enough to accurately detect the object of interest in all cases [13]. Further, traditional methods may exhibit sensitivity to alternations in the visual characteristics of the target object, such as variations in lighting conditions or perspective shifts, which can consequently result in false positive detection [15].

In contrast, deep learning-based object detection techniques exhibit superior speed and efficiency while concurrently addressing the limitations associated with traditional computer-vision-based detection methodologies. Neural networks are core components of deep learning [16]. There are various types of neural networks used for different purposes, such as feedforward neural networks (FNNs) for regression and binary classification [16], recurrent neural networks (RNNs) for language modeling and speech recognition [16], and convolutional neural networks (CNNs) for feature extraction, image recognition, and computer vision tasks [17]. Most modern deep learning models for object detection are mainly based on convolutional neural networks (CNNs) [17]. Here, convolutional layers are cascaded, one after another, to extract features from raw inputs. Each convolutional filter is a small matrix of weights that is used to detect a specific pattern or feature in the input data. For example, a convolutional filter might be designed to detect horizontal edges in an image. As the filter is sliding over the input data, it looks for correlations between the values in the filter and the values in the input data. When it finds a strong correlation, it produces a high value in the corresponding position in the output feature map. This process is repeated for every position in the input data, producing a feature map that encodes the presence of the pattern or feature that the filter is designed to detect [18]. Multiple filters can be applied to the input data, each designed to detect a different pattern or feature. For example, a CNN might have one set of filters for detecting edges, another set for detecting corners, and another set for detecting textures. These filters are learned from data during the training process, allowing the CNN to acquire knowledge about the crucial patterns that hold significance for the given task [18].

An object detection network mainly consists of two subnetworks. The first one is called the feature extraction subnetwork, or backbone network, and the remaining one is known as the detection subnetwork. There are many state-of-the-art backbone or feature extraction networks, like Alexnet [19], VGG16 [20], VGG19 [21], Googlenet [22], Resnet18 [23], Resnet50 [23], and Darknet53 [24]. Among feature extraction CNN models, Resnet50 is distinguished by its superior accuracy. Resnet50 comprises 50 convolutional layers, which, while contributing to its high performance, can also be susceptible to the “dying relu” problem, which arises when the input to the relu activation function is negative, resulting in zero output. If this happens repeatedly for a neuron, it can effectively “die”, ceasing to contribute to the learning process. This phenomenon can hinder the network’s ability to learn complex features, particularly in deep networks like Resnet50. If a feature extraction CNN model were developed with fewer convolutional layers and without experiencing the “dying relu” problem, it could potentially outperform Resnet50 when used as a backbone network.

State-of-the-art detection networks include region-based convolutional neural networks (RCNNs) [25], Fast RCNN [26], Faster RCNN [27], You Only Look Once (YOLO) [28], YOLOv2 [29], YOLOv3 [24], and Single-Shot Detector (SSD) [30]. An RCNN and its variants involve multiple stages of object detection. First, they generate region proposals, then classify each proposal and refine the bounding boxes. This multistep process makes RCNN models relatively slow and unsuitable for real-time applications. The training process for RCNN models can be more cumbersome. The original RCNN requires training multiple models separately (feature extraction, SVM classifier, and bounding box regression). Fast RCNN and Faster RCNN simplify this to some extent, but the overall training pipeline remains more complex than YOLO or SSD. In contrast, YOLO and SSD are known as single-stage object detectors. SSD utilizes multiple feature maps at different scales to handle objects of different sizes. This approach adds complexity to the network. Although, SSD uses multiple feature maps to detect objects of various sizes, it can still struggle with very small objects, as the lower resolution of some feature maps might not capture fine details effectively. The newer versions of YOLO (i.e., YOLOv3 and YOLOv4) have improved small object (such as drones) detection by incorporating multiscale predictions and feature pyramids [31]. These versions introduce more complex architectures, including additional layers and advanced feature pyramids with improved bounding box prediction. If YOLOv2 can be modified to better detect small objects, such as drones, and this modified version demonstrates comparable performance to YOLOv3 and YOLOv4 while retraining much lower complexity, then this modified YOLOv2 would be preferable over the more complex YOLOv3 and YOLOv4 models. This would also make the model more suitable for deployment in resource-constrained environments, such as mobile devices or embedded systems.

## 3. Dataset Preparation

The accessibility of publicly available drone datasets is presently limited in practice because of some privacy concerns, financial constraints, and safety considerations. The utilization of drones is predominantly concentrated within specific industry domains, mostly the military, search-and-rescue operations, and delivery services, which makes the data collection more specific and limited.

**Figure 1:**
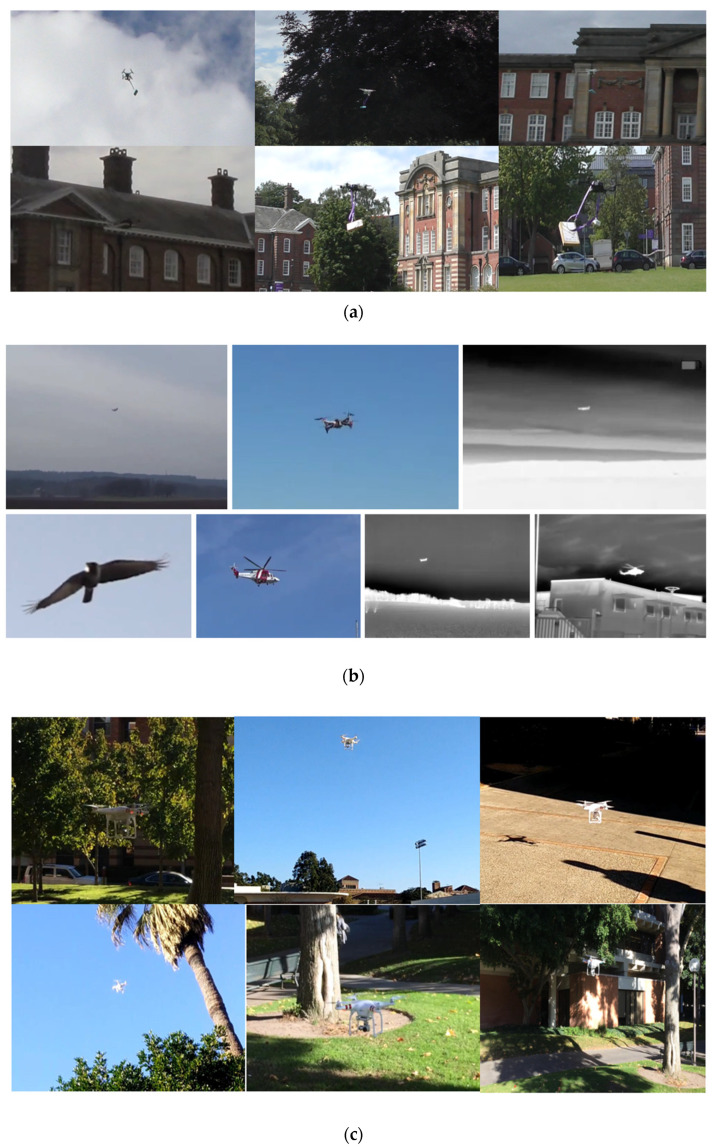
(**a**) Sample frames from the newly created dataset; (**b**) sample frames from the Multi-sensor Drone dataset; (**c**) sample frames from the USC Drone dataset.

A novel drone dataset has been created at Leeds Beckett University (LBU), focusing on three prominent drone models, namely, DJI Phantom (DJI, Nanshan, China), Yuneec (Yuneec, Kunshan, China), and DJI Mavic Mini, as shown in Figure 1a. The acquisition of video footage was executed using the following two distinct camera systems: the Canon LEGRIA HF R806 (Tokyo, Japan), boasting an impressive 32× optical zoom and a focal length ranging from 2.8 mm to 89.6 mm, and the Panasonic Ultra HD (Panasonic, Kadoma, Japan), equipped with a 20× optical zoom and a focal length spanning from 4.08 mm to 81.6 mm. These two cameras are placed in such a way that they always make a triangle with the object of interest. Having two cameras covering different angles helps to mitigate occlusion issues, ensuring continuous tracking even when the object is partially observed from one camera’s perspective. Moreover, combining the views of two cameras can result in a wider field of view, capturing more details and contextual information around the object. This newly created drone dataset is a diverse collection, containing three distinct drones with various loads. After recording the videos, the files were segmented by specifying the time intervals. The new dataset contains 30 video clips of 25 s in length.

The Multi-sensor Drone dataset stands as a monumental repository within the research landscape, representing the most expansive compilation of aerial entities, inclusive of drones and analogous objects, such as airplanes, birds, and helicopters. Offering unfettered access to researchers, this dataset serves as an invaluable resource for the exploration and analysis of various aerial phenomena; samples of datasets frames are shown in Figure 1b [32]. Within the Multi-sensor Drone dataset, a comprehensive collection of aerial entities is encompassed, featuring the three distinct drone models, a small variant represented by the Hubsan H107D+ (Hubsan, Walnut, CA, USA), a midsized counterpart exemplified by the DJI Flame Wheel configured in a quadcopter arrangement, and a high-performance model epitomized by the DJI Phantom 4 Pro [32]. Moreover, this dataset encompasses various flying objects that may be mistakenly identified as drones, like birds, airplanes, and helicopters. The Multisensor Drone dataset comprises a total of 650 video recordings, differentiated into 365 infrared (IR) and 285 visible (RGB) segments, each lasting 10 s [32]. This collective repository yields a corpus of 203,328 meticulously annotated frames, facilitating comprehensive analysis and evaluation of drone classification algorithms [32].

The USC Drone Dataset comprises 30 video recordings captured within the confines of the University of Southern California campus, all filmed utilizing a single drone model; samples of dataset frame are shown in Figure 1c. These recordings were meticulously curated to encompass a diverse array of background scenes, varying camera angles, distinct drone configurations, and diverse weather conditions [33]. Intentionally crafted to encapsulate real-world scenarios, the videos aim to portray the nuances of drone behavior amidst dynamic environmental factors, including rapid motion, challenging lighting conditions, and occlusion phenomena [33]. Each video clip spans an approximately one minute duration, with a frame resolution of 1920 × 1080 and a frame rate set at 15 per second [33].

For training of the CNN models, the new dataset along with the Multi-sensor Drone dataset [32] and USC Drone dataset [33] were used. The inclusion of multiple object classes in the training dataset served to enhance the model’s capacity to discriminate between drones and other similar objects, such as birds and small aircraft. This increases the robustness of the proposed algorithm, making it better able to handle real-world scenarios. When using more classes, the model can learn more features that are useful for classifying different types of objects. The custom dataset consisted of 3000 images of drones, 2000 images of airplanes, 2000 images of helicopters, and 1000 images of birds. This substantial quantity of images proves to be advantageous for training various deep learning models [34].

## 4. Proposed Algorithm

**Figure 2:**
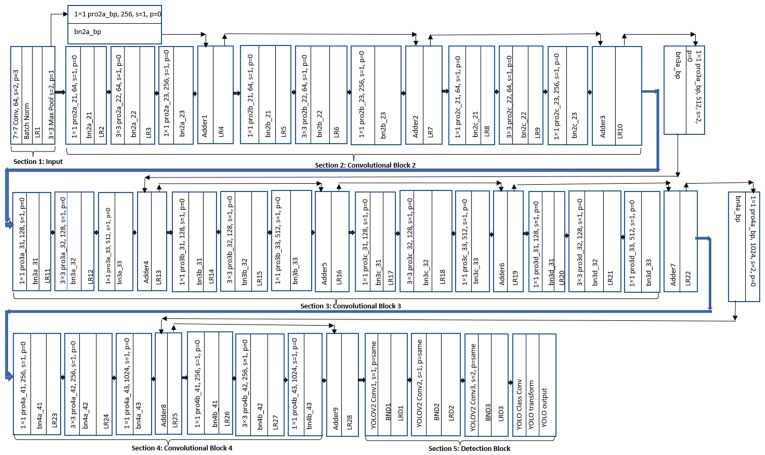
Flowchart of the new algorithm.

The proposed algorithm, as depicted in the flowchart in Figure 2, is structured into five distinct sections, namely, input block, convolutional block 2, convolutional block 3, convolutional block 4, and detection block. Initially, an RGB image is fed into the input block. This block is designed to accept the input dimension of 224 × 224 × 3. Here, the first two dimensions (224 × 224) represent the width and height of the input image, while the last dimension (3) signifies the number of color channels of the image (red, green, and blue). The choice of input size 224 × 224 × 3 was deliberate, as it strikes a balance between being small enough to fit into memory, which is crucial for training large models, and containing sufficient information for tasks such as image classification and object recognition. The dimension is also standard across many well-known datasets, such as the “Imagenet Dataset”. By adhering to this standard input size, researcher can facilitate comparisons between their model and others trained on the same dimensions, fostering a more robust evaluation framework.

The input block consists of one convolutional layer (Conv), one batch normalization layer (batch norm, or BN), one leaky-relu (LR) activation layer, and one max pooling layer. The Conv layer has 64 filters with dimensions of 7 × 7. State-of-the-art feature extraction models usually use the first Conv layer with filter sizes of 3 × 3, 5 × 5, 7 × 7, and 11 × 11. The proposed model works better using 7 × 7 filters at the very initial stage of the network. Using 3 × 3 or 5 × 5 filter at the very first layer causes some contextual information loss. On the other-hand, 11 × 11 filter sources have some extra contextual information, which may not be that useful and destroy the harmony of the feature map reduction. The output of the first 7 × 7-sized Conv with stride of 2 and padding of 3 is 112 × 112 × 64. Thus, the feature map is downsampled to half from its original input dimensions. Batch normalization normalizes the output, calculating the mean and standard deviation of the activations for each channel across the mini-batch. The leaky-relu activation function makes the training smooth. The max pooling layer with a window size of 3 × 3 and stride of 2 is used to further downsample the feature maps and helps to extract information from the more intense pixels. The output of the max pool layer is 56 × 56 × 64.

The convolutional block 2, after the input block, contains three subblocks, namely, 1. proposed convolutional subblock 2a (Pro2a); 2. proposed convolutional subblock 2b (Pro2b); and 3. proposed convolutional subblock 2c (Pro2c). Pro2a has three Conv layers, which are denoted as Pro2a_21, Pro2a_22, and Pro2a_23. After each Conv layer, there is one batch norm layer and one leaky-relu activation layer. Pro2a_21 uses 64 filters with dimensions of 1 × 1 with stride of 1 and padding of 0. Pro2a_22 utilizes 64 filters with sizes of 3 × 3, and Pro2a_23 has 256 filters with sizes of 1 × 1 with the same stride and padding. The first 1 × 1 Conv layers allow the network to learn a more compact representation of the input data, which can be used for the subsequent layers in the network. After that, 64 filters sized 3 × 3 are used, because this is a small enough size to capture the fine-grained details in the image while also being large enough to capture the overall structure of the image. Using a 3 × 3 filter allows the network to learn more complex features than it would with larger or smaller filter dimensions. Additionally, using filters with dimensions of 3 × 3 helps to reduce the number of parameters in the model, making it more efficient and easier to train. Again, 256 filters sized 1 × 1 are used to increase the channel dimensions from 64 to 256 and to trace more details for the feature maps. The output of the input block is directly connected to the output of the pro2a_23 block using “bypass connection”. This bypass connection allows the model to learn more complex and diverse features, improves its ability to relate new features on test data to the most likely features that it learned from the training dataset, and reduces the risk of overfitting. As the dimensions of the feature maps are not the same, 256 filters sized 1 × 1 are used along with the bypass connection. This makes the dimensions of the feature maps and the number of channels equal. This can help the model to learn spatial hierarchies, which can enable it to better understand the relationships between different parts of the feature maps. The pro2b_21, pro2b_22, pro2b_23, pro2c_21, pro2c_22, and pro2c_23 have the same configuration as the pro2a series, apart from having bypass connections without a 1 × 1 Conv layer. For the pro2b series, the 1 × 1 convolutional layers in this configuration are used to reduce the dimensionality of the input, allowing the network to process it more efficiently. The 3 × 3 convolutional layers are used to learn the residual function, and the final 1 × 1 convolutional layer is used to restore the dimensionality of the output. The same is applicable for the pro2c series. By doing so, the model can learn efficiently. Convolutional block 3 and convolutional block 4 have similar connections and configurations. The number of filters is increased or decreased following the power of 2 to maintain harmony. This makes the network more scalable, since it is easy to double the number of filters if more capacity is needed without having to change the overall architecture of the network. The leaky-relu function is used as the activation function throughout the whole network. The leaky-relu is a variant of the relu activation function that allows for a small, nonzero gradient when the input is negative. This helps to prevent the dying relu problem, where many neurons in the network end up outputting a constant zero value and, therefore, stop learning. This improves the model’s performance. The final output of the feature extraction model, which is taken from the output of the “LR28”, is connected to the modified YOLO V2 (YOU ONLY LOOK ONCE VERSION2) detector network. The modified YOLOv2 (mYOLOv2) comprises four subblocks. To incorporate the features from the proposed backbone network into YOLOv2, the first Conv layer in mYOLOv2 serves as a bridge between the two models. The first and second Conv blocks predict the “object-ness” score. The third Conv layer has a stride of 2; hence, it performs the dimensional reduction of the feature maps along with object-ness score perdition. This third sub-block helps to increase the accuracy and robustness by increasing the receptive field, spatial hierarchy, and translation invariance to learn more discriminative features. The YOLO class Conv layer is responsible for predicting the class probabilities for each detected bounding box. The YOLO transform layer helps in transforming the intermediate feature maps into an appropriate form for object detection. The YOLO output layer generates the final predictions by predicting the bounding box coordinates and class probabilities. The default YOLO v2 detector uses the relu function as its activation layers. Here, all default relu activation layers are replaced by leaky-relu activation layers. This brands the detection network more robust to the issue of dying relu units and achieves a better performance in detecting drones.

## 5. Experimental Results

In this paper, a novel CNN-based feature extraction network along with modified YOLO v2 is proposed for detecting drones and similar objects. The performance of the proposed model is compared with state-of-the-art object detection models. The datasets described in Section 3 are used in this experiment.

### 5.1. Proposed Feature Extraction Network with Different Optimizers

The proposed feature extraction network is trained with different optimizers maintaining other hyperparameter constants, like a learning rate of 0.01, max epoch of 10, mini-batch size of 20, and frequency of 50. Table 1 shows that after completing the maximum number of epochs, the mini-batch accuracy was 100%, validation accuracy was 85.14%, mini-batch loss was 0.0069, and validation loss was 1.6661 for the ADAM Optimizer. It can be observed from Table 2 that the mini-batch accuracy was 100%, validation accuracy was 94.86%, mini-batch loss was 0.0001, and validation loss was 0.1034 for the SGDM Optimizer after completing the maximum number of epochs. Hence, the SGDM performed better than the ADAM optimizer with the custom dataset. This is because ADAM maintains adaptive learning rates for each parameter individually, which can introduce noise. The custom dataset used for the training contains many small object images. This means they will have many zero entries. In handling such types of datasets, the SGDM performs better. Moreover, the SGDM can help the update process and prevent overfitting by using momentum [35].

Furthermore, the custom dataset that was used here is full of various objects, like airplanes, birds, drones, and helicopters, of different sizes and aspect ratios. Sometimes ADAM’s adaptive learning rates might not adapt optimally across all dimensions. This could result in suboptimal convergence. The SGDM, on the other hand, tends to work better in such scenarios due to the momentum term, which helps steer the optimization process along the dominant directions and overcome issues related to high dimensionality [35]. Sometimes, ADAM’s adaptive learning rate might struggle to adapt efficiently if the gradients computed during the training process have high variance. SGDM, with its momentum term, can help mitigate this issue and provide more stable convergence [35].

### 5.2. State-of-the-Art Feature Extraction Models vs. Proposed Model

Resnet18, Resnet50, and Darknet53 are considered among the best performing feature extraction networks due to their innovative architectures, which address critical challenges in deep learning. Resnet18, with 18 layers, balances depth and computational efficiency, while Resnet50, with 50 layers, captures more complex features, leading to higher accuracy on challenging datasets. Darknet53 combines a residual network with densely connected layers to optimize both feature extraction and computational efficiency, making it particularly effective for real-time applications.

**Figure 3:**
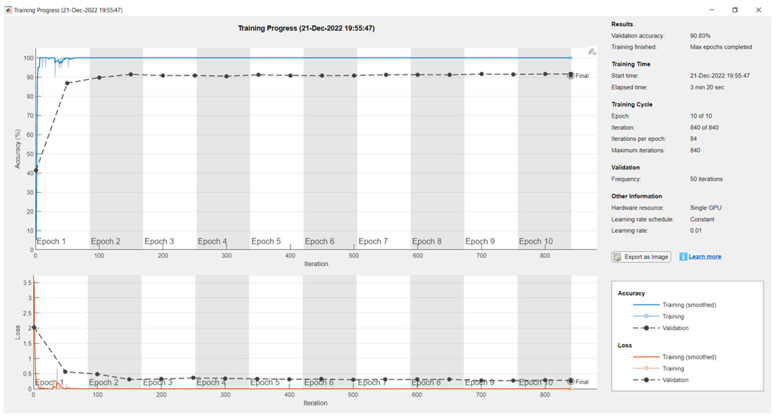
Training and validation results for Resnet18 on the custom dataset.

Figure 3 shows that Resnet18 achieved a 90% validation accuracy with a max epoch of 10, mini-batch size of 20, learning rate of 0.01, and the frequency of 50 on the custom dataset. The elapsed time was 3 min and 20 s. The total learnable properties were 11.1 million.

**Figure 4:**
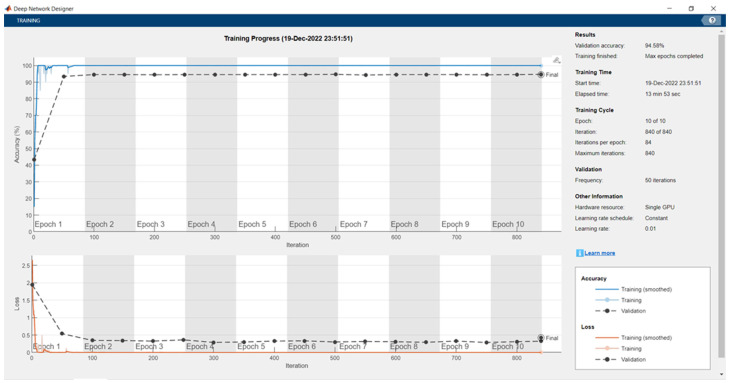
Training and validation results for Resnet50 on the custom dataset.

Figure 4 illustrates that Resnet50 resulted in a 93.75% validation accuracy on the custom dataset with a max epoch of 10, mini-batch size of 20, learning rate of 0.01, and frequency of 50. The elapsed time was 13 min and 53 s. Here, the learnable properties were 23.5 million.

**Figure 5:**
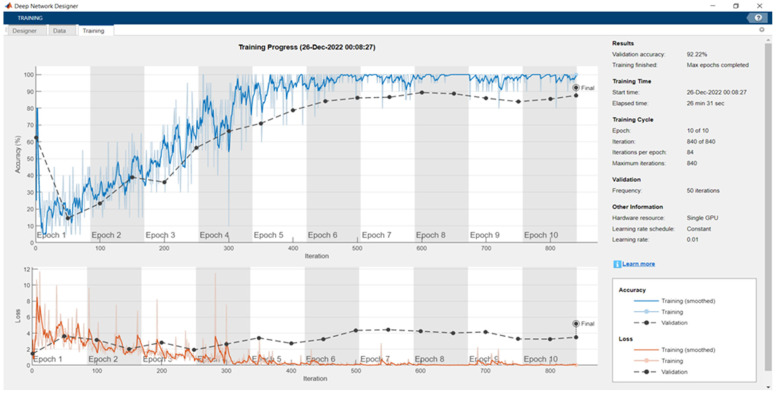
Training and validation results for Darknet53 on the custom dataset.

Figure 5 depicts the training outcomes of the pretrained network Darknet53 with a max epoch of 10, mini-batch size of 20, frequency of 50, and learning rate of 0.01 on the custom dataset. The validation accuracy was below 80% up to an epoch number of 5, becoming stable after epoch number 6, and achieving a 92.22% accuracy at the end with an elapsed time of 26 min 31 s, with almost 40 million learnable properties, and high validation loss.

**Figure 6:**
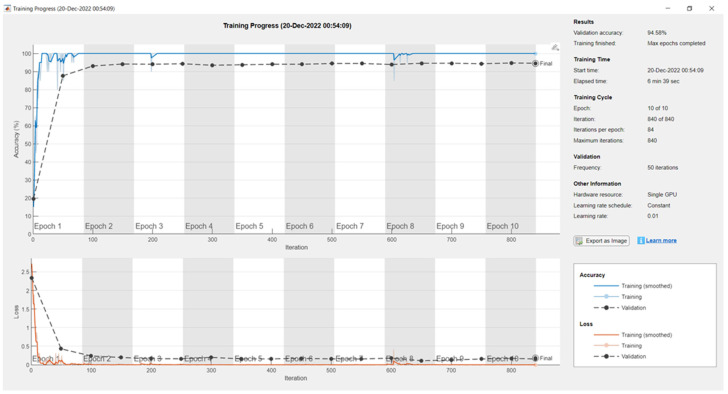
Training and validation results of the proposed feature extraction network on the custom dataset.

Figure 6 portrays that the proposed backbone network for feature extraction yielded a 94.58% validation accuracy on the custom dataset with a max epoch of 10, mini-batch size of 20, learning rate of 0.01, and frequency of 50. The elapsed time needed was 6 min and 39 s. The total learnable properties were 4 million.

The proposed feature extraction method achieved 5% more validation accuracy with almost three times less learnable properties compared to Resnet18. In comparison with Resnet50, the proposed model achieved a 0.85% better accuracy spending half the elapsed time and with almost six times fewer learnable properties. Darknet53 is four times slower than the proposed network. In terms of accuracy and loss, the proposed backbone network attained better results than the Darknet53 with 10 times fewer learnable properties.

**Figure 7:**
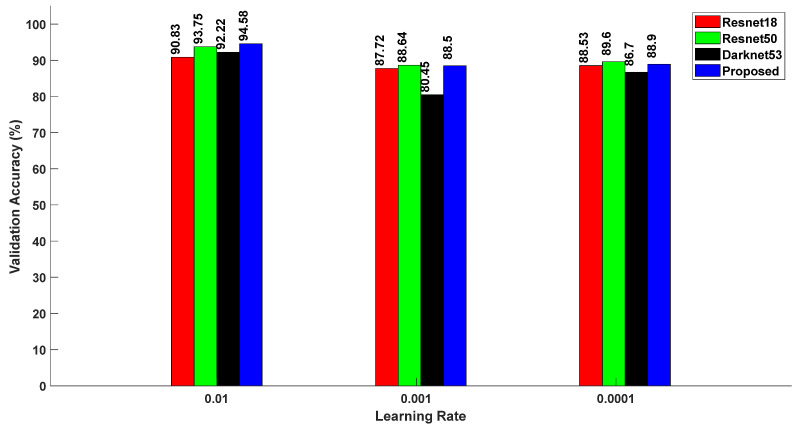
Relationship between validation accuracy and learning rates for various CNN models.

In the conducted experimentation shown in Figure 7, three distinct learning rates were used, namely, 0.01, 0.001, and 0.0001. Each learning rate was associated with a set validation accuracy, corresponding to the following four different CNN architectures: Resnet18, Resnet50, Darknet53, and the proposed method. Specifically, for a learning rate of 0.01, the validation accuracies for the architectures were found to be 90.83%, 93.75%, 92.22%, and 94.58%, respectively. Subsequently, for a learning rate of 0.001, the validation accuracies were recorded as 87.72%, 88.64%, 80.45%, and 88.50% for Resnet18, Resnet50, Darknet53, and the proposed method, respectively. Finally, employing a learning rate of 0.0001 resulted in validation accuracies of 88.53%, 89.60%, 86.70%, and 88.90% for the respective CNN architectures.

As the duration of the learning rate increased, the validation accuracy decreased across all of the specified CNN models. For a learning rate of 0.01, the proposed method achieved a validation accuracy of 94.58%, outperforming the other architectures. The elapsed time variation associated with the specific learning rates were consistently similar across the mentioned experiments, thereby rendering a separate discussion unnecessary.

**Figure 8:**
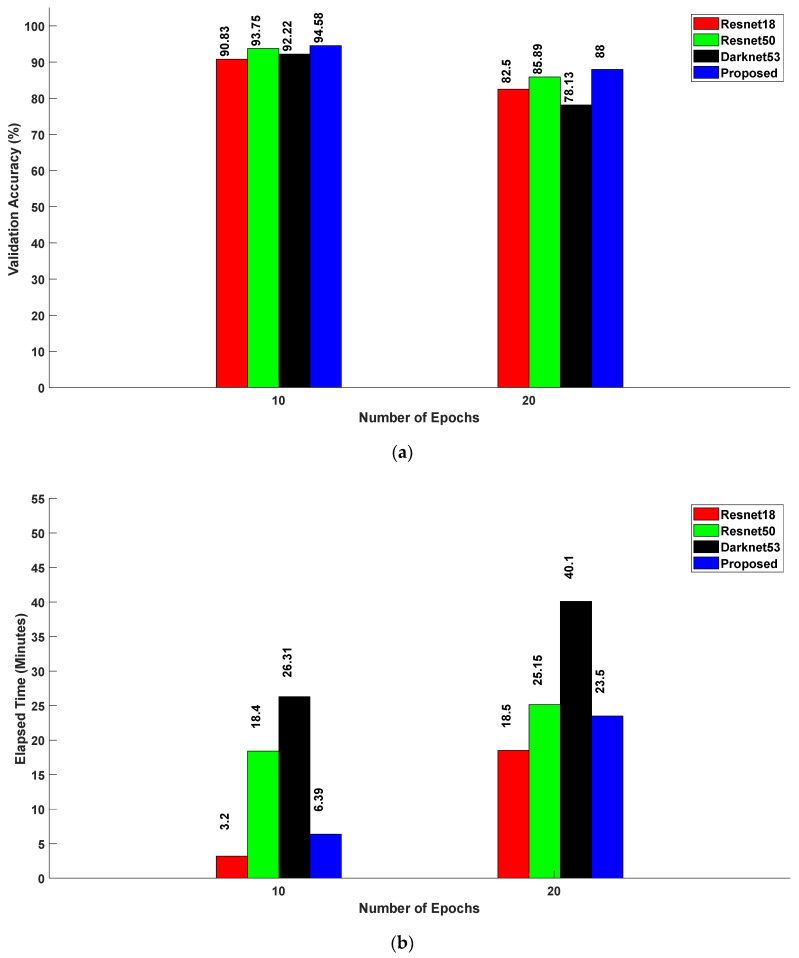
(**a**) Relationship between validation accuracy and number of epochs for various CNN models; (**b**) relationship between elapsed time and number of epochs for various CNN models.

Figure 8a,b correlate the relationship among the validation accuracy, elapsed time, and number of epochs across different neural network architectures, namely, Resnet18, Resnet50, Darknet53, and the proposed method. For 10 epochs, the validation accuracies achieved were 90.83% for Resnet18, 93.75% for Resnet50, 92.22% for Darknet53, and 94.58% for the proposed method. The corresponding elapsed time for these models were 3 min 20 s, 18 min 40 s, 26 min 31 s, and 6 min 39 s. Remarkably, the proposed method consistently exhibited the highest validation accuracy of 94.58% with an elapsed time of 6 min 39 s, beating Resnet18, Resnet50, and Darknet53 when the number of epochs was set to 10. Similarly, at 20 epochs, the validation inaccuracies were 82.50%, 85.89%, 78.13%, and 88%, while the corresponding elapsed times were 18 min 50 s, 25 min 15 s, 40 min 10 s, and 23 min 50 s for Resnet18, Resnet50, Darknet53, and the proposed method, respectively. Once again, the proposed method demonstrated a superior performance, achieving the highest validation accuracy of 88%.

**Figure 9:**
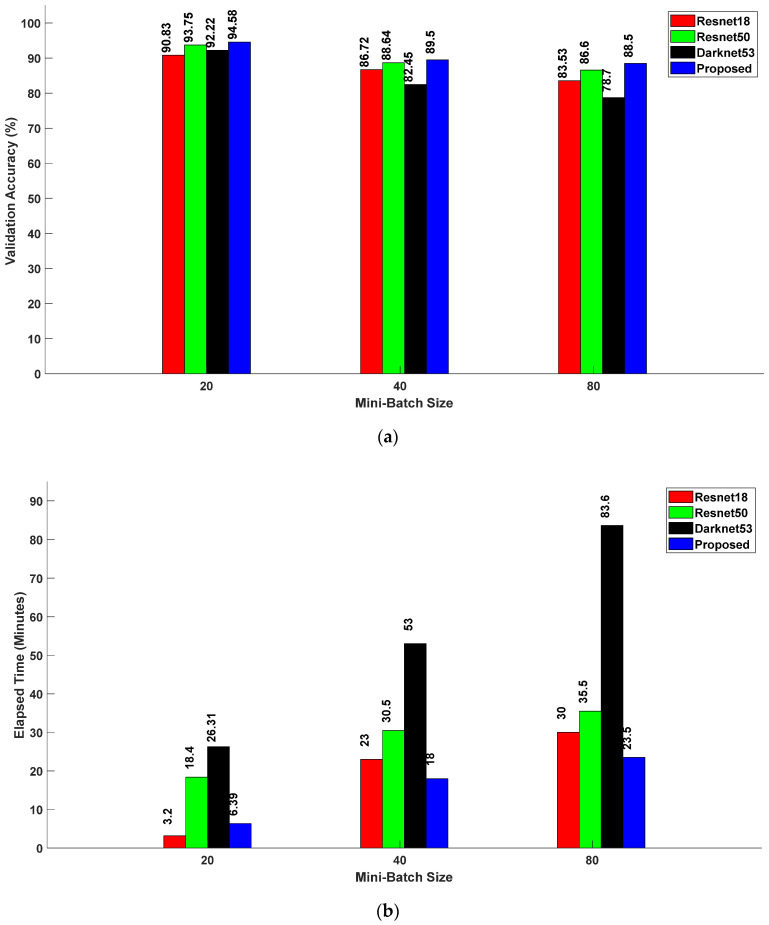
(**a**) Relationship between validation accuracy and mini-batch size for various CNN models; (**b**) relationship between elapsed time and mini-batch size for various CNN models.

Figure 9a,b provide an integrated exploration of the dynamics among validation accuracy, elapsed time, and mini-batch size across distinct neural network architectures, namely, Resnet18, Resnet50, Darknet53, and the proposed method. Resnet18, Resnet50, Darknet53, and the proposed method achieved validation accuracies of 90.83%, 93.73%, 92.22%, and 94.58%, respectively, with their associated elapsed times of 3 min 20 s, 18 min 40 s, 26 min 31 s, and 6 min 39 s. Similarly, with a mini-batch size of 40, the validation accuracies of Resnet18, Resnet50, Darknet53, and the proposed method were recorded as 86.72%, 88.64%, 82.45%, and 89.50%, respectively. The corresponding elapsed times for these models were 23 min, 30 min 50 s, 53 min, and 18 min, respectively. Finally, for a mini-batch size of 80, the validation accuracies were 83.53%, 86.60%, 78.70%, and 88.50%, while the associated elapsed times were 30 min, 35 min 50 s, 83 min 60 s, and 23 min 50 s for Resnet18, Resnnet50, Darknet53, and the proposed method, respectively. Consistently, the proposed method outperformed the other architectures, emphasizing its superior performance across varying mini-batch sizes.

**Figure 10:**
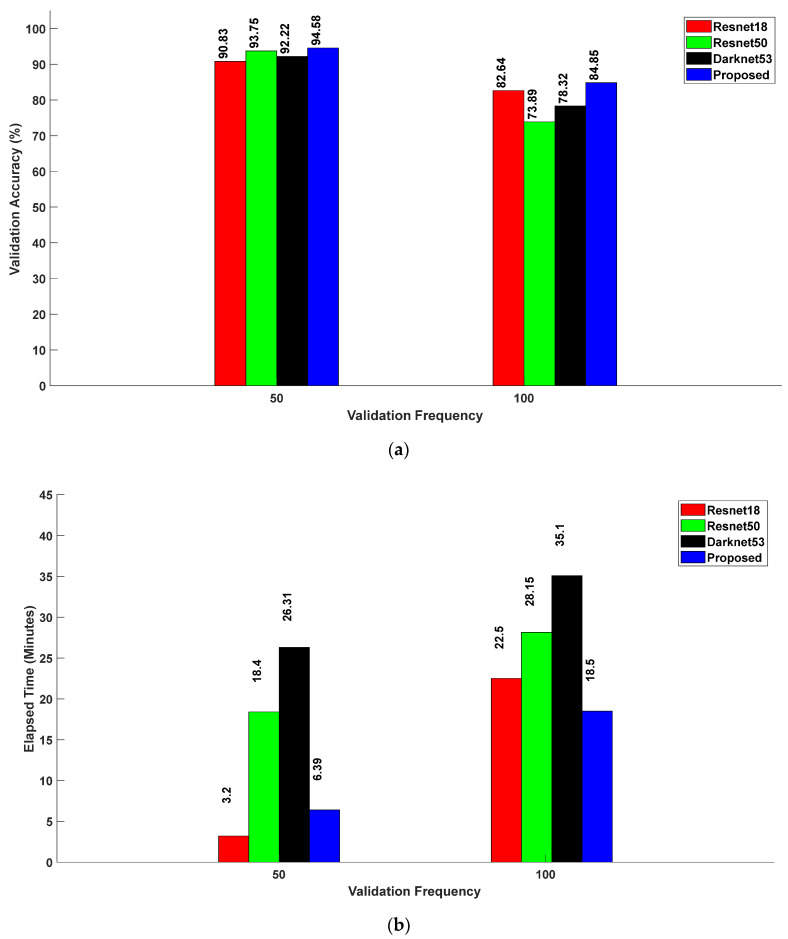
(**a**) Relationship between validation accuracy and validation frequency for various CNN models; (**b**) relationship between elapsed time and validation frequency for various CNN models.

Figure 10a,b present comparative analyses of the validation accuracies and associated elapsed times for Resnet18, Resnet50, Darknet53, and the proposed method at validation frequencies of 50 and 100. With a validation frequency of 50, the validation accuracies of Resnet18, Resnet50, Darknet53, and the proposed method were 90.83%, 93.75%, 92.22%, and 94.58%, respectively. The associated elapsed times were 3 min 20 s, 18 min 40 s, 26 min 31 s, and 6 min 39 s, respectively. The proposed method achieved the highest validation accuracy of 94.58% with an elapsed time of 6 min 39 s. When the validation frequency was increased to 100, the validation accuracies were 82.64% for Resnet18, 73.89% for Resnet50, 78.32% for Darknet53, and 84.85% for the proposed method. The corresponding elapsed times for these models were 22 min 50 s, 28 min 15 s, 35 min 10 s, and 18 min 50 s, respectively. Similarly, with a frequency of 100, the proposed method yielded the highest validation accuracy of 84.85% with the smallest elapsed time of 18 min 50 s, outperforming Resnet18, Resnet50, and Darknet53.

### 5.3. Anchor Boxes Estimation

Accurately estimating the number of anchor boxes is a key factor in designing a detector that can achieve superior performance. The Intersection over Union (IoU) calculation helps to determine the similarities between the predicted boxes and the ground-truth boxes, thus enabling the network to learn how to localize object’s precisely. The IoU is given by (1), as follows [36]:

(1) $$IoU=\frac{Overlap\;area\;between\;bounding\;box\;of\;predicted\;and\;ground\;truth\;}{Combine\;area\;between\;bounding\;box\;of\;predicted\;and\;ground\;truth}$$

**Figure 11:**
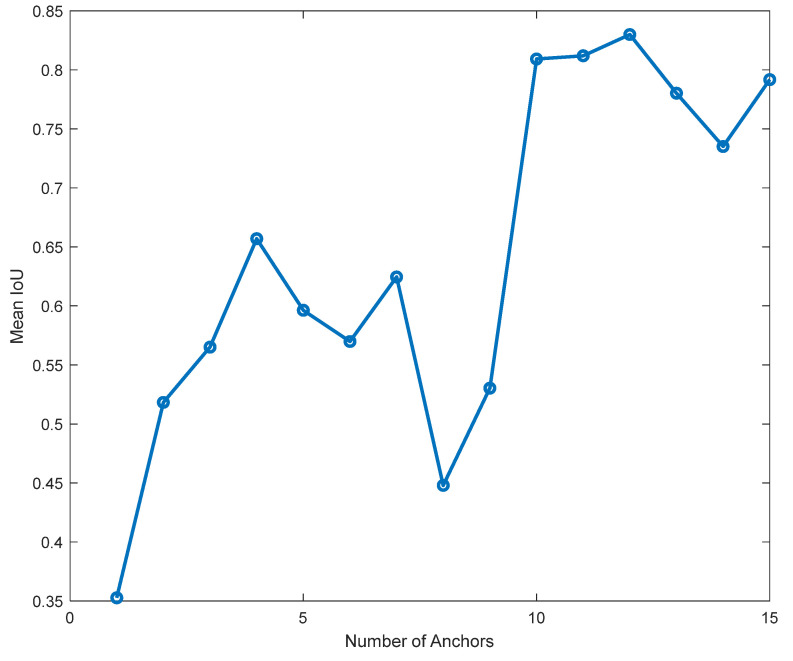
Number of anchor vs. mean IoU curve.

A threshold is set to determine whether a predicted box is a good match to the ground truth. If the IoU between the predicted box and ground-truth box exceeds the threshold, it is considered a positive match. Figure 11 describes the relationship between mean IoU and number of anchor boxes for the custom dataset. It is observed that the highest mean IoU (Mean IoU = 0.839996) was obtained when the number of anchor boxes was 12. This means the best number of anchors to increase the detector performance is 12.

### 5.4. Modified YOLOv2 Network with Different Optimizers

**Figure 12:**
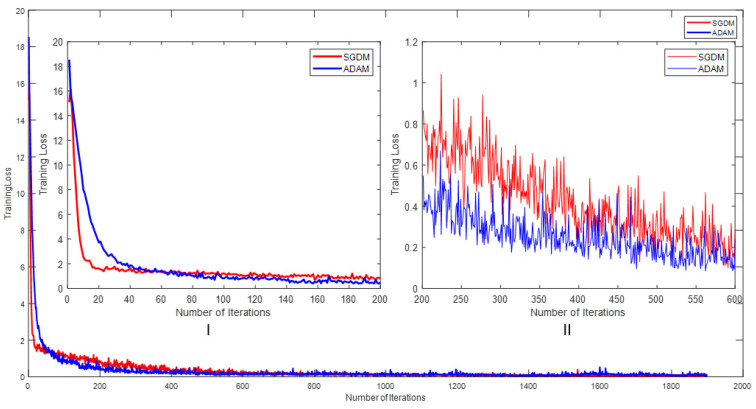
Number of iterations vs. training losses for different optimizers.

The training losses with respect to the number of iterations for the different optimizers are displayed in Figure 12. Figure 12I provides a zoomed-in view of iterations 0 to 200, while Figure 12II focuses on iterations 200 to 600. It can be observed that the initial losses for the SGDM and ADAM were 15.0906 and 18.5401, respectively. The SGDM’s training losses were better than ADAM’s when the number of iterations were from 0 to 50. When the number of iterations crossed 50, ADAM had relatively better results than the SGDM, and this continued up to 500. After, the SGDM and ADAM exhibited almost the same performances. Hence, for a detection network, either the SGDM or ADAM can be used, as both had similar performances with the custom dataset.

### 5.5. State-of-the-Art Feature Extraction Models with YOLOv2 vs. Proposed Feature Extraction Model with Modified YOLOv2

In this section, state-of-the-art feature extraction networks, namely, Resnet50, Resnet18, and Darknet53, were added to the You Only Look Once version2 (YOLOv2) detector and trained with the custom dataset. After the training, they were tested with the test dataset. At the same time, the proposed feature extraction network was combined with the modified YOLOv2 detector and trained on the custom dataset. The proposed new model for feature extraction with the modified YOLOv2 detector was applied to the test dataset to verify its performance.

#### 5.5.1. Resnet18 + YOLOv2

**Figure 13:**
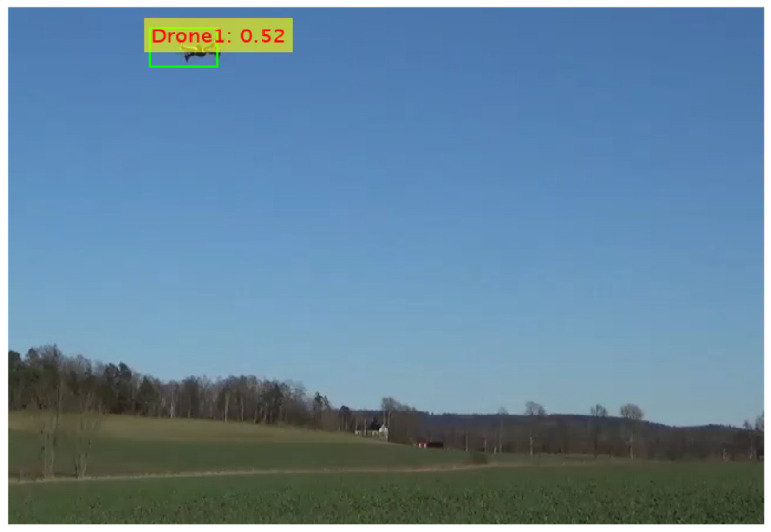
Confidence score for YOLOv2 with Resnet18.

The Resnet18 feature extraction model was integrated with the YOLOv2 (You Only Look Once version2) object detection framework which was shown in Figure 13. The time needed to train the detector was 89 min and 12 s with a max epoch of 20, max iterations of 1900, iterations per epoch of 95, and learning rate of 0.001. The total learnable properties were 15.9 million. Upon completion of the training phase, the model was evaluated using test images. The evaluation results indicate that the model effectively detected drones, achieving a confidence score of 52%.

#### 5.5.2. Resnet50 + YOLOv2

**Figure 14:**
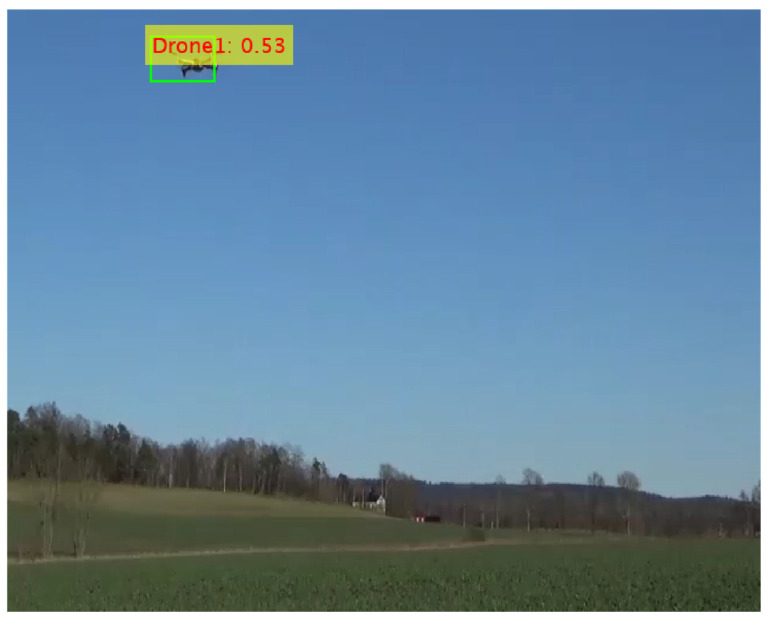
Confidence score for YOLOv2 with Resnet50.

Figure 14 illustrates the model’s confidence in detecting objects in the test images when the Resnet50 feature extraction network was combined with the YOLOv2 object detection framework. The training process for the detector encompassed a duration of 82 min and 48 s employing the following specific parameters: max epoch of 20, max iterations of 1900, iterations per epoch of 95, and learning rate of 0.001. The model comprised 27.5 million learnable parameters. Following the training phase, the model was evaluated using test images. The evaluation shows that the model detected drones with a confidence score of 53%.

#### 5.5.3. Darknet53 + YOLOv2

**Figure 15:**
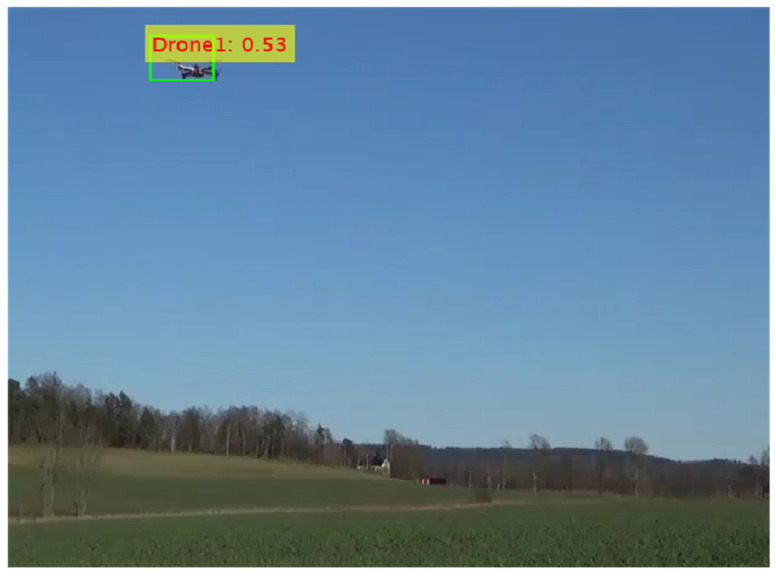
Confidence score for YOLOv2 with Darknet53.

Figure 15 depicts the confidence levels of the model in identifying objects in test images, utilizing the Darknet53 feature extraction network in conjunction with YOLOv2 object detection framework. The training process for the detector spanned 91 min 30 s, utilizing the following parameters: a maximum of 20 epochs, 1900 total iterations, 95 iterations per epoch, and a learning rate of 0.001. The total learnable parameters were 41.60 million. After training, the performance of the model was tested on the test images. The model was able to detect drones with a confidence score of 53%.

#### 5.5.4. Darknet53 + YOLOv3

**Figure 16:**
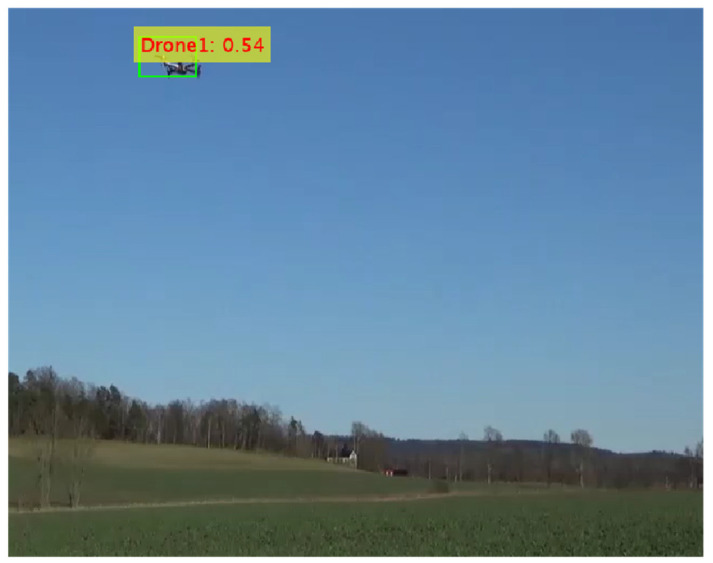
Confidence score for YOLOv3 with Darknet53.

Figure 16 shows the confidence scores for object detection in test images, achieved by integrating the Darknet53 feature extraction network with the YOLOv3 object detection framework. The training time required for the YOLOv3 detector was 130 min with a max epoch of 20, max iterations of 1900, iterations per epoch of 95, and learning rate of 0.001. In the parallel pooling, eight workers worked simultaneously. This model had a total of 62 million learnable parameters. The model detected drones with a confidence score of 53%.

#### 5.5.5. Proposed + Modified YOLOv2

**Figure 17:**
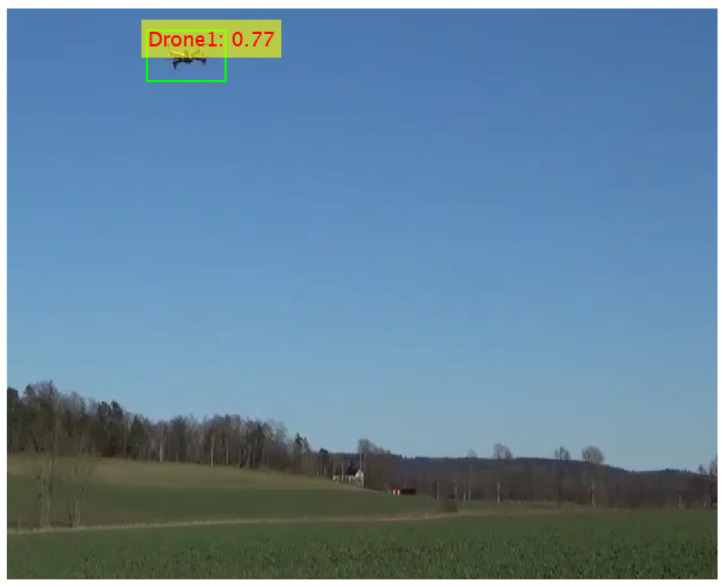
Confidence score for the modified YOLOv2with the proposed backbone network.

Figure 17 displays the accuracy levels of the proposed model (proposed backbone network + modified YOLOv2) at identifying objects in test images. The time needed to train the new model was about 80 min and 19 s, with a max epoch of 20, max iterations of 1900, iterations per epoch of 95, and learning rate of 0.001. The total quantity of learnable parameters amounted to 5 million. The model demonstrated an ability to detect drones and similar objects with a confidence score of 77%.

Table 3 presents various models along with their respective quantities of learnable parameters and corresponding test accuracy rates. The relationships between model complexity, quantified as the number of learnable parameters, and performance is a focal point of discussion. Typically, increased complexity, as indicated by a higher number of learnable parameters, heightens the risk of overfitting. Overfitting occurs when a model excessively tailors itself to the training data, impairing its ability to generalize effectively to unseen data.

In contrast, an optimal model often strikes a balance between complexity and performance. This balance is reflected in the model being characterized by fewer learnable parameters yet achieving a higher test accuracy. For instance, the proposed model, with a modest 5 million learnable parameters exhibited a notably high accuracy in test detection of 77%. This suggests that despite its relatively lower complexity compared to the other models, the proposed model effectively captures the underlying patterns in the data and generalizes well to new instances.

Conversely, models with higher complexities, such as Darknet53 + YOLOv3, with 62 million learnable parameters, may encounter greater challenges in generalization despite their extensive capacity to represent complex patterns. The observed performance variations across models underscores the intricate interplay between model complexity and predictive capability, emphasizing the importance of striking an optimal balance in designing effective deep learning architectures.

## 6. Performance Evaluations

Precision–recall curves (PR curves), area under the precision–recall curves (AUC-PR), and F1 score threshold curves were considered in evaluating the performance of the state-of-the-art models. Precision is the number of true positive detections divided by the total number of positive detections [37]. On the other hand, recall is the number of true positive detections divided by the total number of actual detections [37]. The F1 score is the trade-off between Precision and Recall.

(2) $$Precision=\frac{True\;Positive}{True\;Positive+False\;Positive}$$

(3) $$Recall=\frac{True\;Positive}{True\;Positive+False\;Negative}$$

(4) $$F1Score=\frac{2\times Precision\times Recall}{\left(Precision+Recall\right)}$$

If a model has high precision and low recall, this means the model is very selective about what it considers to be positive detections. So, it will only output a positive detection if it is very confident about the positive class detection. The problem is that due to its high selectiveness it might not detect all object classes in an image. On the other hand, a model with high recall and low precision is less selective in detecting positive classes. So, it will output many objects as a positive class object with a low confidence score. It might detect all objects that are in the image but will result in many false object class detections. The performance of any model is better if it can maintain a high precision value when the recall is increasing from lower to higher values [38]. The area under the precision–recall curve (AUC-PR) is another important measurement of how well a model works. The higher the area under the PR curve, the better the model [38].

**Figure 18:**
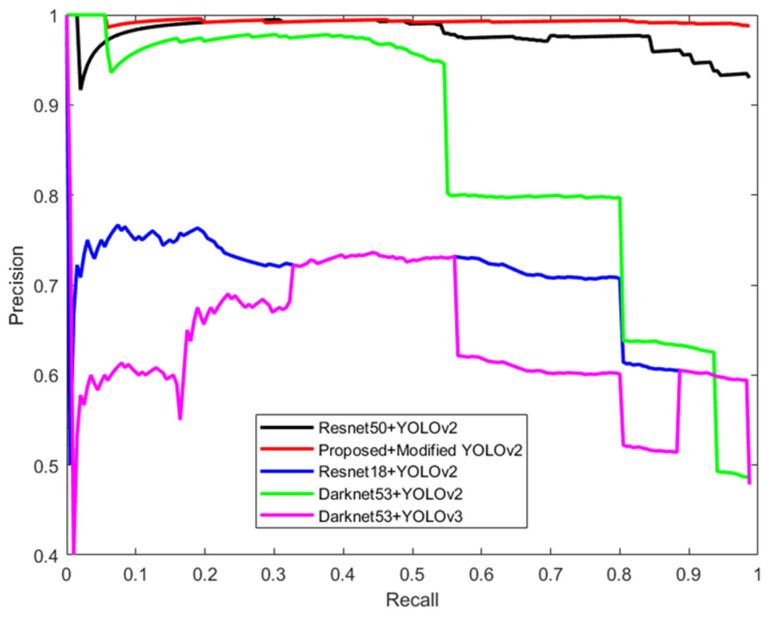
Precision–recall curves of the proposed model and state-of-the-art models for drone classes.

Figure 18 shows the precision–recall (PR) curves of various state-of-the-art models alongside the proposed model. An analysis of these curves reveals that the precision curve of the proposed model consistently maintains higher values compared to the other models as the recall increases from 0 to 1. Notably, the precision curve for the Resnet50 + YOLOv2 is slightly lower than the proposed model for recall values ranging from 0 to 0.24. For recall values between 0.24 and 0.5, the precision curves of both the proposed model and Resnet50 + YOLOv2 overlap. Beyond a recall value of 0.5, the proposed model consistently outperformed Resnet50 + YOLOv2, demonstrating superior precision. In comparison, the precision curves for Resnet18 + YOLOv2 and Darknet53 + YOLOv2 were significantly lower than that of the proposed model.

Further, the Darknet53 + YOLOv3 model exhibited the lowest area under the precision–recall curve. This performance discrepancy is attributed to the more complex architecture of YOLOv3, which may result in higher rates of false positives and false negatives, particularly with custom datasets. While YOLOv3 excels in multiscale object detection, it may encounter challenges in precise location under certain conditions, thereby affecting the PR curve. Overall, the proposed model demonstrated the largest area under the PR curve compared to the state-of-the-art models evaluated. This indicates that the proposed model offers superior performance in terms of the AUC-PR metric.

**Figure 19:**
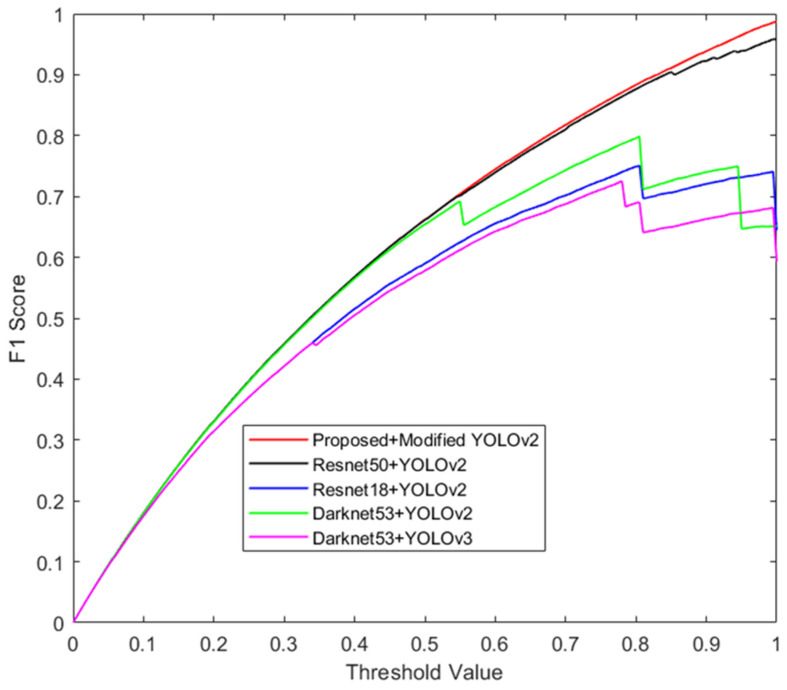
F1 score–threshold curves of the proposed model and state-of-the-art models for drone classes.

Another evaluation criterion is the F1 score. The F1 score is a measurement of a model’s accuracy that combines precision and recall. It is defined as the harmonic mean of the precision and recall. The F1 score evaluates the performance of a model by considering both the ability of the model to correctly detect objects (i.e., recall) and its ability to avoid false detection (i.e., precision) [39]. Figure 19 portrays the F1 score curves for different state-of-the-art models and the proposed model with respect to threshold values. The F1 score curves of the proposed model (proposed + modified YOLOv2) and the Resnet50 + YOLOv2 overlapped from 0 to 0.85. Beyond the threshold value of 0.85, the proposed model remains on top, ahead of Resnet50 + YOLOv2. The F1 curves of Resnet18 + YOLOv2 and Darknet53 + YOLOv2 stay well below the proposed model. The lowest area under the F1 curve is covered by the Darknet53 + YOLOv3 model. The F1 score curve spans a larger area if a model’s hyperparameters have the right alignment with the dataset. In this respect, the modifications made to the original YOLOv2 with the new feature extraction network has this alignment with the custom dataset. The F1 score curve of the proposed model had the largest area under the curve compared to the state-of-the-art models.

## 7. Discussion

As presented in Section 2, a novel dataset featuring drones equipped with various payloads was curated to facilitate the detection of drones operating within constrained environments, such as airport and prisons, with a particular focus on identifying drones transporting illicit substances and explosive materials. The newly assembled drone dataset was combined with two publicly accessible datasets, namely, “Multi-sensor Drone Dataset” and “USC Drone Dataset”. The custom dataset comprised 8000 images of drones alongside analogous objects such as airplanes, helicopters, and birds. The dataset’s size proves notably advantageous for convolutional neural networks (CNN)-based models, striking a balance between adequacy for robust model training and testing without being overly small or excessively large.

The proposed model comprises a modest architecture consisting of 31 convolutional layers, strategically integrated with “bypass connections” to address the prevalent vanishing gradient encountered in deep neural networks. By facilitating alternative pathways for gradient flow during backpropagation, these bypass connections enable earlier layers to directly access activations from deeper layers. Consequently, this architectural design promotes the efficient reuse of features learned at varying depths of the network, enhancing the model’s capacity to learn diverse and robust representations of input data. Additionally, the incorporation of the leaky-relu activation function throughout the network effectively mitigates the “dying relu” problem, ensuring the consistent propagation of information across layers. Moreover, an augmentation of the architecture included the incorporation of a new convolutional layer, featuring a stride of 2, in the detection block. This augmentation was coupled with the sequential addition of a batch normalization layer and a leaky-relu activation layer. This comprehensive modification enhanced the detector’s capabilities to detect small objects from images and video frames.

The proposed model, configured with specific hyperparameters and optimizer settings, demonstrates utility when applied to a dataset characterized by noisy information and objects with diverse aspect ratios. However, for lower levels of noise, adjustments to the configuration may be necessary for optimal performance.

The efficacy of the proposed model is further accentuated by its careful management of learnable parameters, which are pivotal in shaping the model’s efficiency, complexity, and susceptibility to overfitting. Through a deliberate balance between efficiency and complexity, the proposed model showcases a remarkable effectiveness while mitigating the risk of overfitting. Notably, despite its modest composition of 31 convolutional layers and 5 million learnable parameters, the proposed model achieved a noteworthy 77% on the test images, highlighting its proficiency in capturing intricate patterns and exhibiting robust generalization capabilities across unseen instances. Hence, the proposed model is more suitable for resource-constrained environments, such as mobile devices or embedded systems.

## 8. Conclusions

**Figure 20:**
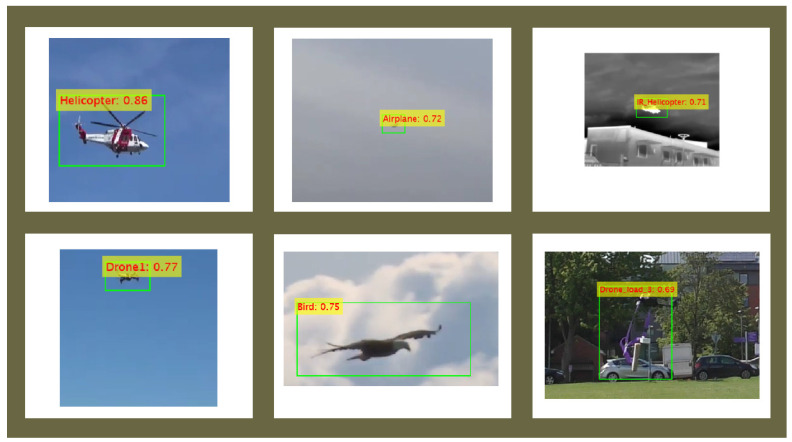
Outcomes for the proposed detection model.

In this paper, dominant state-of-the-art algorithms for object detection were applied to a custom dataset. The experiment’s results show that the new proposed feature extraction network with the modified YOLOv2 detector achieved better accuracy with less elapsed time and fewer learnable properties compared to existing state-of-the-art models. The proposed model resulted in a 77% confidence score in detecting drones from the test dataset, whereas the Darknet53 + YOLOv3 model achieved 54%. The proposed model is very robust as it was trained on multiple classes of objects, like drones, airplanes, helicopters, and birds, under various weather conditions. Consequently, with highly dense backgrounds containing numerous similar objects, as shown in Figure 20, the proposed model detects a “drone with Load3” with a confidence score of 69%. This result exemplifies a high level of performance. The constant high precision value when recall increased from 0 to 1, as well as the area under the precision–recall curve and the F1 score–threshold curve, ensures that the performance of the proposed model outperforms state-of-the-art models. In summary, the proposed model demonstrates a paradigm shift in convolutional neural network design, demonstrating that efficacy and efficiency need not sacrificed in favor of complexity and learnable properties.

## Figures and Tables

**Table 1:** Training and validation based on ADAM.

Epoch	Iteration	Elapsed Time (hh:mm:ss)	Mini-Batch Accuracy	Validation Accuracy	Mini-Batch Loss	Validation Loss	Base Learning Rate
1	1	00:00:15	5.00%	25.97%	2.7688	4.2363	0.0100
1	50	00:01:20	75.00%	61.53%	0.5686	1.3813	0.0100
2	100	00:02:26	95.00%	76.53%	0.1081	1.3301	0.0100
2	150	00:03:33	95.00%	73.47%	0.3317	1.1884	0.0100
3	200	00:04:42	100.00%	74.17%	0.04370	1.4640	0.0100
3	250	00:05:50	100.00%	86.53%	0.05330	1.2644	0.0100
4	300	00:06:58	95.00%	67.92%	0.1578	2.3191	0.0100
5	350	00:08:05	100.00%	80.28%	0.0177	1.1865	0.0100
5	400	00:09:12	100.00%	81.94%	0.0088	1.2314	0.0100
6	450	00:10:20	100.00%	86.39%	0.0029	1.1369	0.0100
6	500	00:11:27	100.00%	86.39%	0.0029	1.1369	0.0100
7	550	00:12:36	75.00%	79.31%	0.7070	1.5656	0.0100
8	600	00:13:43	100.00%	85.14%	0.0539	1.3672	0.0100
8	650	00:14:49	90.00%	85.56%	0.3977	1.0454	0.0100
9	700	00:15:24	100.00%	87.64%	0.0427	1.0036	0.0100
9	750	00:15:53	95.00%	90.83%	0.0540	0.9987	0.0100
10	800	00:16:21	100.00%	85.14%	0.0176	2.0756	0.0100
10	840	00:16:45	100.00%	85.14%	0.0069	1.6661	0.0100

**Table 2:** Training and validation based on SGDM.

Epoch	Iteration	Elapsed Time (hh:mm:ss)	Mini- Batch Accuracy	Validation Accuracy	Mini-Batch Loss	Validation Loss	Base Learning Rate
1	1	00:00:09	0.00%	23.97%	2.7197	2.4006	0.0100
1	50	00:00:46	100.00%	93.19%	0.0028	0.1610	0.0100
2	100	00:01:23	100.00%	94.72%	0.0017	0.1380	0.0100
2	150	00:02:02	100.00%	93.19%	0.0096	0.2071	0.0100
3	200	00:02:39	100.00%	95.97%	0.0054	0.0916	0.0100
3	250	00:03:17	100.00%	95.97%	0.0011	0.0932	0.0100
4	300	00:04:07	100.00%	96.11%	0.0004	0.0998	0.0100
5	350	00:05:08	100.00%	95.28%	0.0016	0.0908	0.0100
5	400	00:06:09	100.00%	96.11%	0.0002	0.1004	0.0100
6	450	00:07:11	100.00%	95.28%	0.0004	0.1023	0.0100
6	500	00:08:14	100.00%	95.69%	0.0003	0.1136	0.0100
7	550	00:09:17	100.00%	95.42%	0.0014	0.1045	0.0100
8	600	00:10:19	100.00%	96.11%	0.0001	0.1116	0.0100
8	650	00:11:24	90.00%	94.72%	0.0006	0.1101	0.0100
9	700	00:12:27	100.00%	94.86%	0.0001	0.1153	0.0100
9	750	00:13:30	100.00%	95.42%	0.0015	0.1083	0.0100
10	800	00:14:32	100.00%	95.69%	0.0002	0.1211	0.0100
10	840	00:15:25	100.00%	94.86%	0.0001	0.1034	0.0100

**Table 3:** Learnable properties vs. test accuracy of the different models.

Model	Learnable Properties (In Millions)	Test Accuracy
Resnet18 + YOLOv2	15.90	52%
Resnet50 + YOLOv2	27.50	53%
Darknet53 + YOLOv2	41.60	53%
Darknet53 + YOLOv3	62.00	54%
Proposed	5.00	77%

## Data Availability

The data presented in this study are available upon request from the corresponding author. The data are not publicly available due to privacy concerns.
